# Matching Patients to Accelerate Clinical Trials (MPACT): Enabling Technology for Oncology Clinical Trial Workflow

**DOI:** 10.3233/SHTI231132

**Published:** 2024-01-25

**Authors:** Nhan V DO, Danne C ELBERS, Nathanael R FILLMORE, Samuel AJJARAPU, Steven J BERGSTROM, John BIHN, June K CORRIGAN, Rupali DHOND, Svitlana DIPIETRO, Arkadiy DOLGIN, Theodore C FELDMAN, Sergey D GORYACHEV, Linden B HUHMANN, LA Jennifer, Paul A MARCANTONIO, Kyle M MCGRATH, Stephen J MILLER, Vinh Q NGUYEN, George R SCHNEELOCH, Feng-Chi SUNG, Kaitlin N SWINNERTON, Amelia H TARREN, Hannah M TOSI, Danielle VALLEY, Austin D VO, Cenk YILDIRIM, Chunlei ZHENG, Robert ZWOLINSKI, Gisele A. SAROSY, David LOOSE, Colleen SHANNON, Mary T BROPHY

**Affiliations:** a VA Boston Healthcare System, Boston MA, USA; b Harvard Medical School, Boston MA, USA; c Boston University School of Medicine, Boston MA, USA; d National Cancer Institute, Bethesda MD, USA

**Keywords:** Clinical trials, workflow, NLP

## Abstract

Clinical trial enrollment is impeded by the significant time burden placed on research coordinators screening eligible patients. With 50,000 new cancer cases every year, the Veterans Health Administration (VHA) has made increased access for Veterans to high-quality clinical trials a priority. To aid in this effort, we worked with research coordinators to build the MPACT (Matching Patients to Accelerate Clinical Trials) platform with a goal of improving efficiency in the screening process. MPACT supports both a trial prescreening workflow and a screening workflow, employing Natural Language Processing and Data Science methods to produce reliable phenotypes of trial eligibility criteria. MPACT also has a functionality to track a patient’s eligibility status over time. Qualitative feedback has been promising with users reporting a reduction in time spent on identifying eligible patients.

## 1. Introduction

Oncology clinical trials can advance our understanding of the prevention, diagnosis, and treatment of cancer. For patients who fail first line therapy, clinical trials can offer an avenue to novel and potentially life-prolonging treatments. Unfortunately, the success of many trials is hampered by an inability to meet targeted patient accruals [1]. Although multiple causes have been identified, one critical factor is the lack of efficient eligibility screening processes. Identifying patients for clinical trials is time-consuming; Penberthy’s estimate is 6.33–8.78 hours required per patient for enrolling in Phase I-III trials [2].

Multiple strategies using Information Technology (IT) to increase efficiency of trial screening and recruitment processes have been reported [3]. The application of Natural Language Processing (NLP) and machine learning methods to automate the matching of a subset of eligibility criteria such as demographics, staging, biomarkers, and treatment have produced promising results in terms of high sensitivity and specificity [4–6]. However, the limitations of these reports are stated as either single disease, single center, or limited data access and integration. Most importantly these reports do not offer insights into the workflow where these automation tools can be used to maximize their value [3].

The Veterans Health Administration (VHA) has an estimated 50,000 incident cancer cases annually [7]. One of the strategic priorities of the VHA’s Office of Research and Development is “Increase Veterans’ access to high-quality clinical trials.” To facilitate Veterans’ access to oncology clinical trials, the NIH National Cancer Institute (NCI) and the Department of Veterans Affairs (VA) have partnered to create a program known as the NCI And VA Interagency Group to Accelerate Trials Enrollment (NAVIGATE) [8]. The program’s objectives include increasing Veteran participation in NCI-funded clinical trials by building national infrastructures that reduce enrollment barriers and promote sustained patient participation. Together with NAVIGATE research coordinators (RCs), we have identified workflow automation opportunities and built the MPACT (Matching Patients to Accelerate Clinical Trials) application, presented here. MPACT’s principal goal is to reduce the amount of time spent identifying & enrolling eligible patients during oncology trial screening process.

## 2. Methods

Requirement creation, system design, workflow modeling, and usability assessment were conducted together with an RC workgroup. Additional semi-structured interviews and think-aloud sessions were held individually with five coordinators for usability assessment.

Development of MPACT follows a user-centered design approach and Agile method to facilitate rapid build-test-deploy cycles. This includes sequential deployment and testing through development, integration, and production environments, assuring all new versions pass comprehensive quality assurance checks before release. The VA Enterprise GitHub platform is used for software version control, tracking application requirements, and managing both development and deployment cycles.

Data sources for MPACT include the VA’s Corporate Data Warehouse (CDW), VA cancer registry, and the VA National Precision Oncology Program (NPOP) for targeted genomic sequencing results. Data are extracted using R-based data pipelines which run nightly. NLP is used for unstructured data from clinical notes such as ECOG score, cancer stage, and PD-L1 status. MPACT’s graphical user interface (GUI) employs a Python-driven Flask microframework enhanced with Microsoft Active Directory Security access controls.

## 3. Results

Our requirements analysis indicated that there are two main workflows for our development effort: a prescreening workflow and a screening workflow. The main objective in the prescreening workflow is to identify potential patients to screen when they visit the clinic for routine care. Working with RCs, we identified three high-impact opportunities for automation in the prescreening workflow: 1) automated prescreening list, 2) search filters for additional phenotypic and care-related data, and 3) data integration with future clinic appointments. While recognizing that it is not feasible to automate the extractions and mapping of all trial inclusion or exclusion criterion from the medical record, the RCs identified specific, high-value eligibility criteria that could reduce the patient screening list to a perceived manageable number for full screening. These criteria are cancer type and subtype, cancer stage and metastatic status, biomarkers, and prior therapy. Additional filters for a specific trial can be requested by RCs for additional high-value data elements that are available in the Electronic Health Record (EHR). Through integration of near real-time patient appointments, an RC can create a date range for the upcoming week with a clinic selection to screen a list of patients with scheduled appointments.

**Figure 1.**
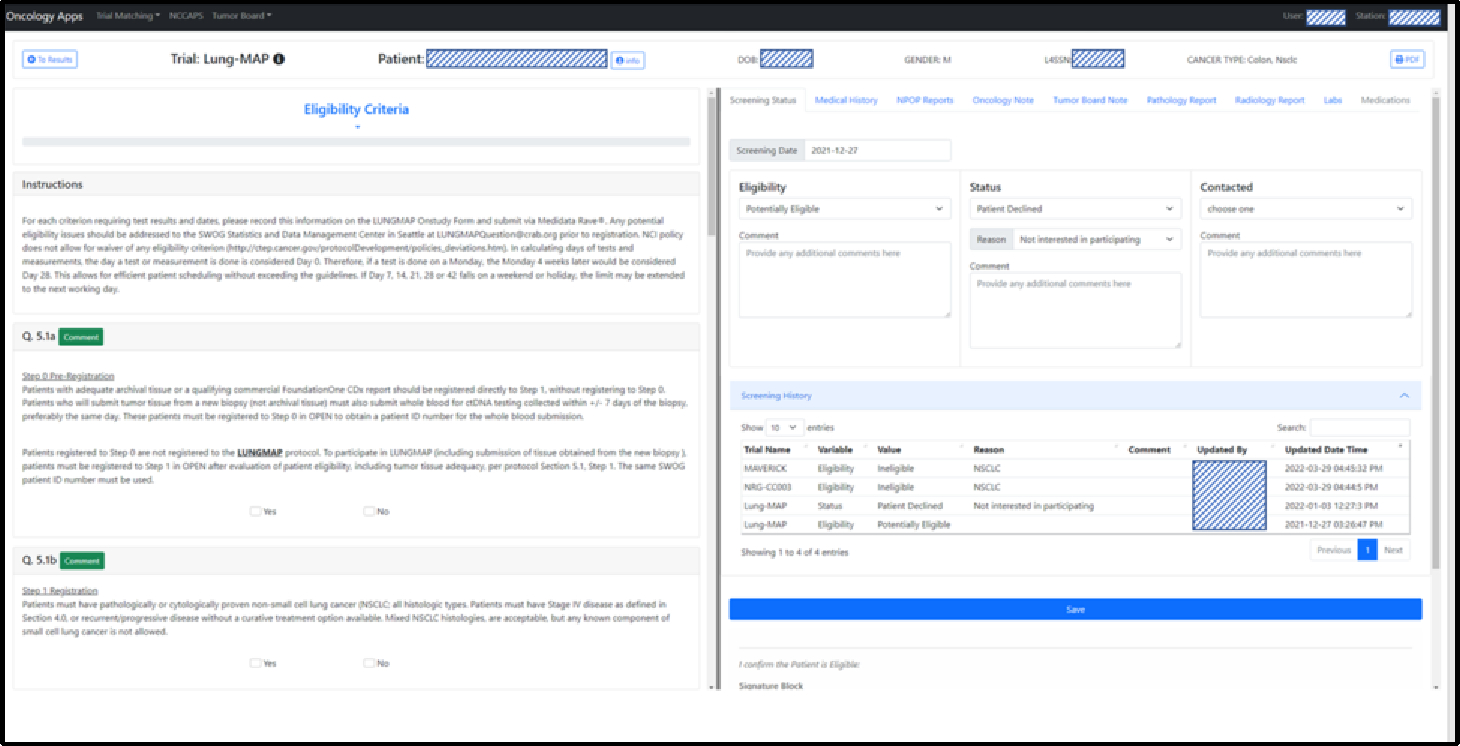
Eligibility Screening.

**Figure 2.**
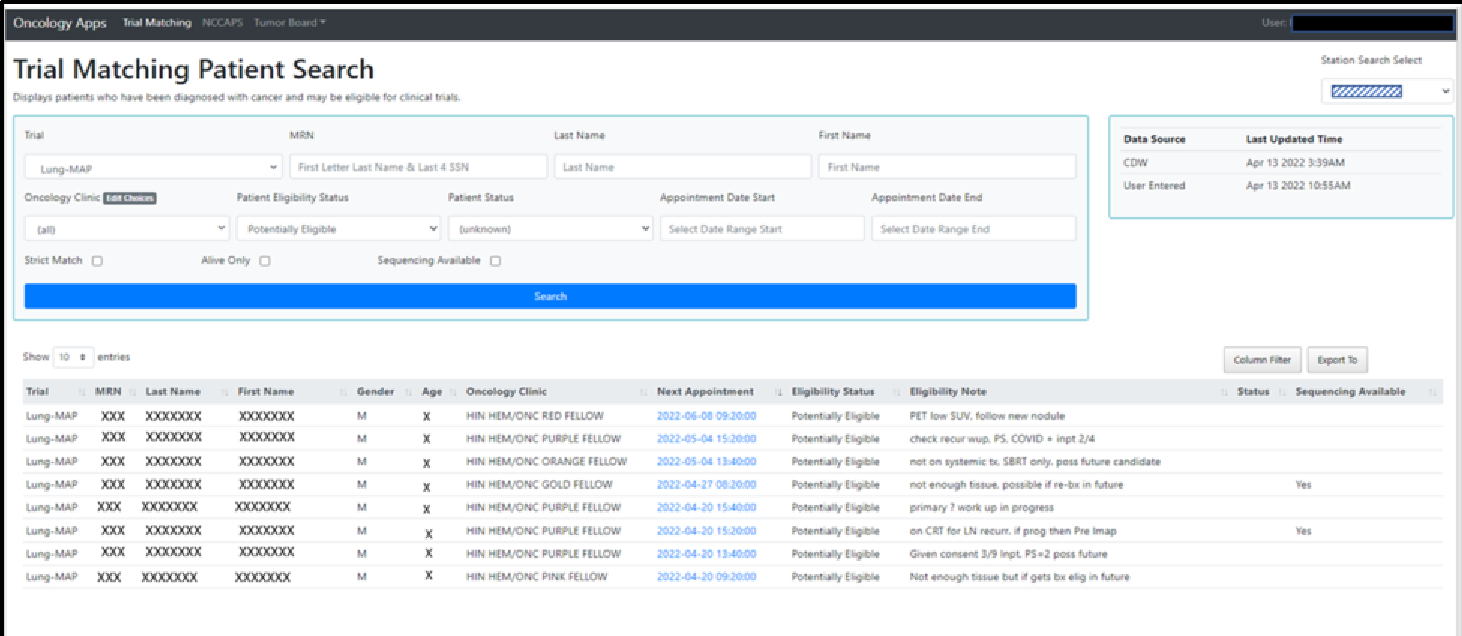
Dashboard tracking potentially eligible patients.

The main objective of the screening workflow is to confirm eligibility criteria against the patient’s health record. We identified three key high-impact opportunities for facilitating the screening workflow: 1) electronic eligibility criteria worksheet, 2) eligibility criteria review, and 3) tracking of screened patients. Figure 1 shows a screen displaying the trial’s inclusion and exclusion criteria side-by-side with the patient’s medical history extracted from the EHR. This functionality allows the RC to quickly eliminate patients who do not meet eligibility criteria. A tracking functionality is provided to monitor eligibility and enrollment status. This functionality facilitates communication among RCs about patients who are not to be approached again because they have either been enrolled, failed screening, or refused screening. Figure 2 shows a dashboard of screened patients with upcoming appointments and pending tasks.

MPACT is currently being piloted with 20 oncology trials (5 prostate, 1 bladder, 1 colon, 1 Chronic Lymphocytic Leukemia (CLL), 1 Chronic Myeloid Leukemia (CML), 10 lung, and 1 head and neck) at 19 VA facilities with 35 active users. One of the main themes from RC interviews was time efficiency. We asked if using MPACT improves screening efficiency and which functionalities produced the most efficiency gain. All participants of the structured interview reported time savings, and one participant quantified this gain as “usually took me 6 hours to do” before MPACT and now “I’ve done screening in under an hour.” This was made possible through use of MPACT’s automated prescreening list integrated with upcoming appointments and the availability of EHR data in one location for quick removal of ineligible patients. Before the implementation of MPACT, patients with rare biomarkers used to repeatedly appear as new screening opportunities. With MPACT’s patient tracking functionality, RCs can avoid “rescreening the same patients over and over again”, reducing RC time burden.

## 4. Discussion

The objective of MPACT is to improve the efficiency of the screening process for oncology trials. After identifying automation opportunities in the prescreening and screening workflow, we developed and piloted a software application to assist VA RCs with oncology trial recruitment. While the literature offers examples of IT tools to support clinical trial enrollment, they do not offer the breadth of implementation for multiple cancer types, number of sites, and the spectrum of workflow support identified by the RCs. Commercial products such as IBM Watson or open-source platforms such as MatchMiner would be limited to the inherent workflow found in these products along with challenges of data mapping and integration. As an example, MatchMiner is well suited for the prescreening workflow but not for the screening workflow [9]. A limitation of this report is that the feedback collected from our group of users is part of an iterative user-centered design process and not a formal qualitative research study. While all RCs reported a reduction of screening time, only one provided a quantity in hours reduced. As MPACT continues to expand to more VA sites and oncology trials, we plan to further evaluate usability, adoptions, and clinical impact.

## 5. Conclusions

MPACT was developed to improve efficiency in the labor-intensive trial eligibility screening process. Our work demonstrates that by focusing data extraction, NLP, and interactive GUI design efforts around key steps within the trial screening workflow, we can move closer to our organization’s goal of increasing our patients’ access to clinical trials.
